# TIMP-1 is upregulated, but not essential in hepatic fibrogenesis and carcinogenesis in mice

**DOI:** 10.1038/s41598-017-00671-1

**Published:** 2017-04-06

**Authors:** Nina D. Thiele, Jan W. Wirth, David Steins, Anja C. Koop, Harald Ittrich, Ansgar W. Lohse, Johannes Kluwe

**Affiliations:** 1 Department of Internal Medicine, University Medical Center Hamburg-Eppendorf, Hamburg, Germany; 2 Department of Diagnostic and Interventional Radiology and Nuclear Medicine, University Medical Center Hamburg-Eppendorf, Hamburg, Germany

## Abstract

Tissue inhibitor of metalloproteinases-1 (TIMP-1) is upregulated during hepatic fibrogenesis and considered to promote fibrosis in the injured liver by inhibition of matrix metalloproteases (MMP) and degradation of extracellular matrix. Moreover, TIMP-1 displays anti-apoptotic properties, in patients with hepatocellular carcinoma (HCC) TIMP-1 serum levels are elevated and high TIMP-1 expression levels in HCC are associated with a poor prognosis. Therefore, TIMP-1 could functionally link fibrogenesis and carcinogenesis in the liver. The aim of our study was to characterize the role of TIMP-1 in hepatic fibrogenesis and carcinogenesis. Experimental hepatic fibrogenesis as well as diethylnitrosamine (DEN) -induced hepatocarcinogenesis were studied in TIMP-1-deficient mice and wild type littermates. Hepatic TIMP-1 expression was upregulated following induction of liver fibrosis by bile duct ligation (BDL) or by carbon tetrachloride (CCl_4_). Unexpectedly, in comparison to wild type littermates, TIMP-1-deficient mice were not protected from liver fibrosis induced by BDL or CCl_4_. TIMP-1 expression was significantly higher in HCC nodules than in surrounding liver tissue. However, experimental hepatic carcinogenesis was similar in TIMP-1-deficient mice and wild type littermates following DEN-treatment or combined treatment with DEN and CCl_4_. Therefore we concluded that TIMP-1 is not essential for hepatic fibrogenesis and carcinogenesis in mice.

## Introduction

Chronic liver injury, irrespective of the underlying disease, triggers a wound healing response that leads to fibrogenesis and can ultimately result in the development of liver cirrhosis. Patients with liver cirrhosis are at risk for mortality by complications caused by portal hypertension and liver failure. Moreover, underlying liver cirrhosis represents a major predisposition for the development of hepatocellular carcinoma (HCC)^1, 2^. Thus, the majority of HCC occurs in an environment which is characterized by fibrogenic cells and mediators. In spite of the unique epidemiologic association between liver cirrhosis and HCC it is still unclear how the cirrhotic microenvironment contributes to hepatic carcinogenesis.

Tissue inhibitor of metalloproteinases 1 (TIMP-1) regulates remodeling of the extracellular matrix (ECM) in the liver by matrix metalloproteases (MMPs)^3^. In response to liver injury, TIMP-1 is expressed as a consequence of the interaction between activated hepatic myofibroblasts and liver macrophages^4^, two major cellular components of the cirrhotic microenvironment. Moreover, TIMP-1 has been found to be strongly upregulated in liver tissue and serum during hepatic fibrogenesis in patients with liver disease and in animal models of hepatic fibrogenesis; its expression directly correlates with the stage of hepatic fibrosis^5–9^. Several functional studies in mice with either transgenic TIMP-1 overexpression or using an antibody or MMP-9 mutants to antagonize TIMP-1 *in vivo* suggest that TIMP-1 in fact promotes hepatic fibrogenesis^10–13^. In contrast, another study demonstrated increased carbon tetrachloride (CCl_4_)-induced liver fibrosis in TIMP-1 deficient mice^14^. Thus, while published evidence for the functional role of TIMP-1 in hepatic fibrogenesis is ambivalent, there is a predominance of data supporting a profibrotic function of TIMP-1 in the injured liver. In line with this, TIMP-1 has been shown to inhibit apoptosis in hepatic stellate cells (HSC)^15^. Moreover, TIMP-1 executes antiapoptotic effects also in lymphocytes and epithelial cells^16, 17^ and overexpression of TIMP-1 in mammary carcinoma cells and in the retina promoted VEGF-mediated angiogenesis^18, 19^. Both, antiapoptotic and proangiogenic effects are often considered as mechanisms of tumor promotion. Clinical data show that elevated TIMP-1 levels in the sera of patients with breast cancer^20^, gastric, colorectal and ovarian carcinoma^21–23^ as well as non-small cell lung cancer^24^ are correlated with poor survival. Importantly, high TIMP-1 serum levels are significantly correlated with the presence of HCC in patients with chronic liver disease^25^. Moreover, a recent study showed that TIMP-1 expression is elevated in HCC tissues compared to adjacent liver tissue and that higher TIMP-1 expression predicted a worse prognosis^26^. In summary, there is extensive evidence for carcinogenic and prometastatic functions of TIMP-1^27, 28^.

Thus, TIMP-1 harbors functions that might contribute to both, hepatic fibrogenesis and carcinogenesis, and has been epidemiologically associated with liver fibrosis and HCC. Therefore, TIMP-1 represents a possible functional link between development of cirrhosis and HCC. TIMP-1 could represent a promising target for prevention or therapy of HCC because experimental strategies of TIMP-1 inhibition *in vivo* have already been established^12, 13^.

Here, we investigate the role of TIMP-1 as a potential link between hepatic fibrogenesis and carcinogenesis in wild type and TIMP-1-deficient mice.

## Results

### Activated Hepatic Stellate Cells from *TIMP-1 ko* mice lack TIMP-1 protein but express alpha-smooth muscle actin

**Figure 1:**
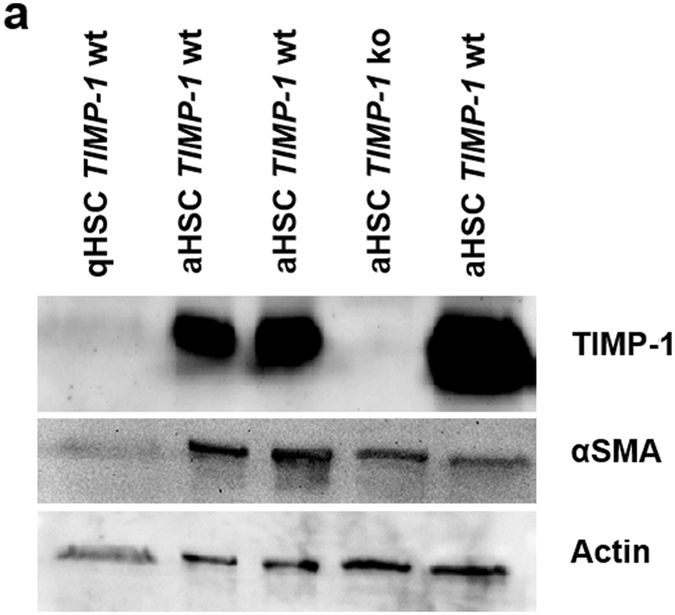
Confirmation of TIMP-1 protein deletion in hepatic stellate cells from *TIMP-1 ko mice*. (**a**) Cropped Western Blots showing no TIMP-1 protein in quiescent stellate cell (qHSC) lysates and activated stellate cells (aHSC) isolated from *TIMP-1* ko mice, whereas activated stellate cells from *TIMP-1* wt livers show elevated TIMP-1 protein level. Activation status of hepatic stellate cells is indicated by αSMA expression. Each lane represents lysed HSCs pooled after isolation from 3 mice. Full-length blots are presented in Supplementary Figure 3.

In the original description of the *TIMP-1* knockout mouse^29^, TIMP-1 deletion had been documented on the nucleic acid level. To confirm deletion of TIMP-1 protein in *TIMP-1 ko* mice, we isolated murine HSCs, a cell population with a high level of TIMP-1 expression following fibrogenic activation^4^. TIMP-1 protein expression was analyzed by Western blot in HSCs from wild type and *TIMP-1 ko* mice. While quiescent wild type HSCs, isolated from normal livers, displayed no TIMP-1 protein expression, *in vivo*-activated HSCs isolated from livers that were injured by CCl_4_ –treatment (4 i.p. injections over two weeks until 48 hours prior to isolation of HSCs), showed a strong induction of the HSC activation marker alpha-smooth muscle actin (αSMA) and a strong TIMP-1 protein signal. No TIMP-1 protein expression was detected in *in vivo* activated HSCs from *TIMP-1 ko* mice, while αSMA expression was preserved (Fig. 1). Thus, deletion of TIMP-1 protein was confirmed in *TIMP-1 ko* mice but no direct effect of TIMP-1-deficiency on HSC activation was documented.

### TIMP-1-deficiency does not prevent hepatic fibrogenesis

It has been well established that TIMP-1 expression is upregulated during liver fibrogenesis^5–8^ and, given the fact that TIMP-1 counteracts proteolytic activity of MMPs, TIMP-1 is generally regarded as a profibrogenic mediator. Quantification of *TIMP-1*-mRNA expression in livers of wild-type mice after fibrosis induction by BDL or CCl_4_ treatment confirmed that TIMP-1 was significantly upregulated during hepatic fibrogenesis in our experimental setting (Supplementary Figure S1).

**Figure 2:**
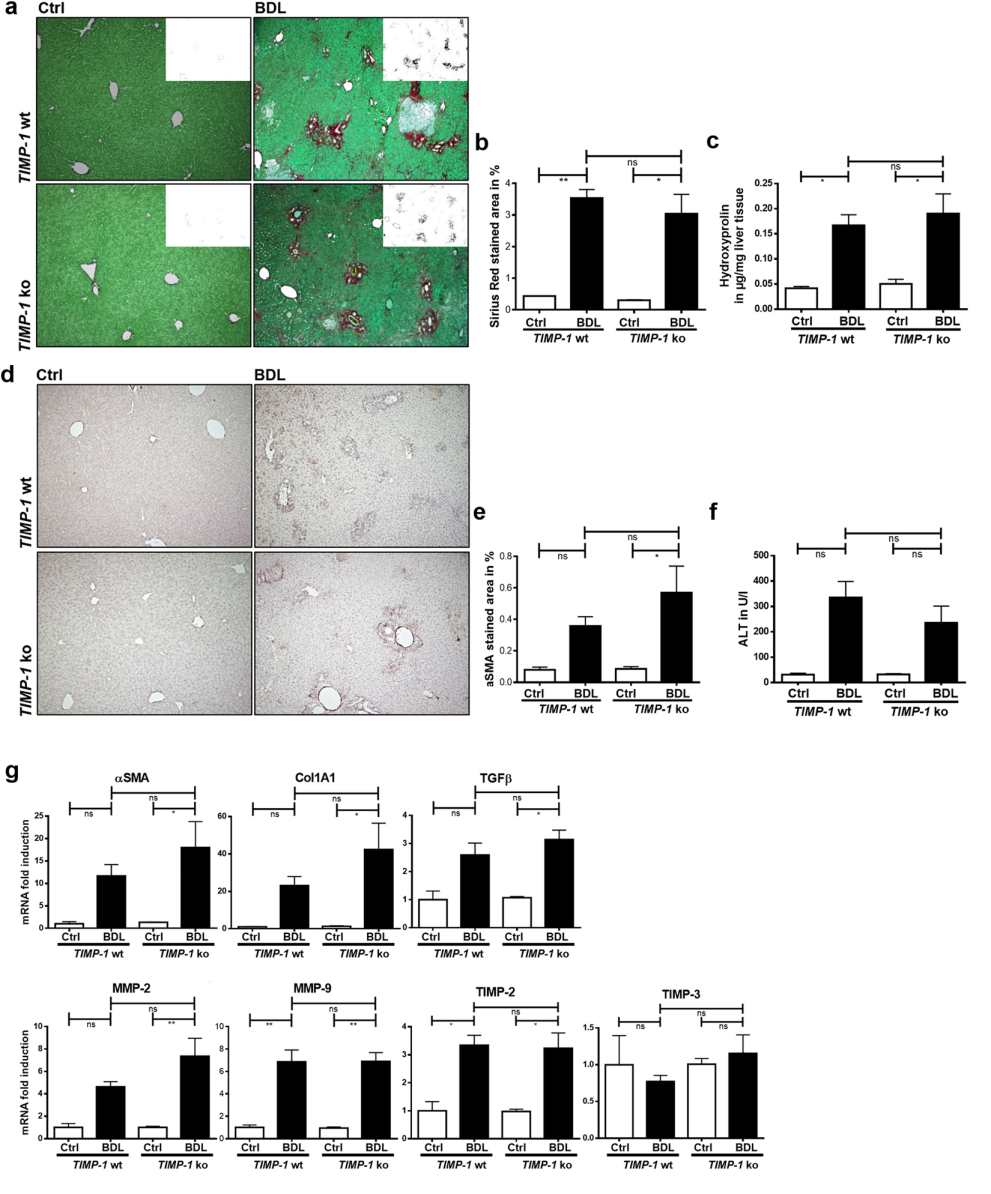
TIMP-1 deficiency does not prevent hepatic fibrogenesis in the BDL model. (**a**) Collagen content of livers 21d post BDL was assessed by Sirius Red staining. TIMP-1 deficiency does not alter Collagen content compared to *TIMP-1* wt, as shown in representative images and in (**b**) quantification of Sirius Red positive stained area. (**c**) Hepatic hydroxyproline content shows also an induction after BDL treatment, but no differences between *TIMP-1* ko and wt. (**d**,**e**) αSMA IHC indicates hepatic stellate cell activation and was increased in livers following 21d of BDL. *TIMP-1* ko have the same amount of αSMA positive cells compared to *TIMP-1* wt. (**f**) Liver transaminases (ALT) were elevated following BDL but did not differ significantly between *TIMP-1 wt* and *TIMP-1 ko*. (**g**) Fibrosis-related genes were upregulated in BDL livers, but no differences could be observed between *TIMP-1* wt and *TIMP-1* ko mice. Bar columns represent mean ± standard error of the mean. **p <  0.01; *p < 0.05; ns (non-significant). (Untreated controls n = 2–4, BDL n = 5–11).

To test the hypothesis that TIMP-1 is in fact functionally relevant for the development of liver fibrosis we subjected wild type and *TIMP-1 ko* mice to two different models of liver fibrosis. Unexpectedly, Sirius Red staining for fibrillary collagen and αSMA immunohistochemistry for activated HSCs revealed no differences in hepatic fibrosis between wild type and *TIMP-1* ko mice following cholestatic liver injury induced by bile duct ligation (BDL) (Fig. 2a–e). These findings are consistently supported by the results of hepatic Hydroxyproline measurement, which showed no difference between *TIMP-1* ko and wildtype mice following BDL (Fig. 2c). Moreover, hepatic mRNA expression of fibrosis-related genes in mice after BDL was not attenuated in TIMP-1-deficient mice (Fig. 2g) in comparison to wild type mice. ALT levels were elevated following BDL but there were no significant differences between wild type and *TIMP-1* ko mice (Fig. 2f). Kupffer cells are associated with inflammation and fibrogenesis in hepatic injury and infiltrate the liver in high numbers following BDL. This is accompanied with upregulation of inflammatory genes like MCP1 (Macrophage Chemoattractant Protein 1) and IL6 (Interleukin 6). TIMP-1-deficiency had no influence on macrophage infiltration into the liver and inflammation following BDL as demonstrated by F4–80 immunohistochemistry and inflammatory gene expression (Supplementary Figure S2).

**Figure 3:**
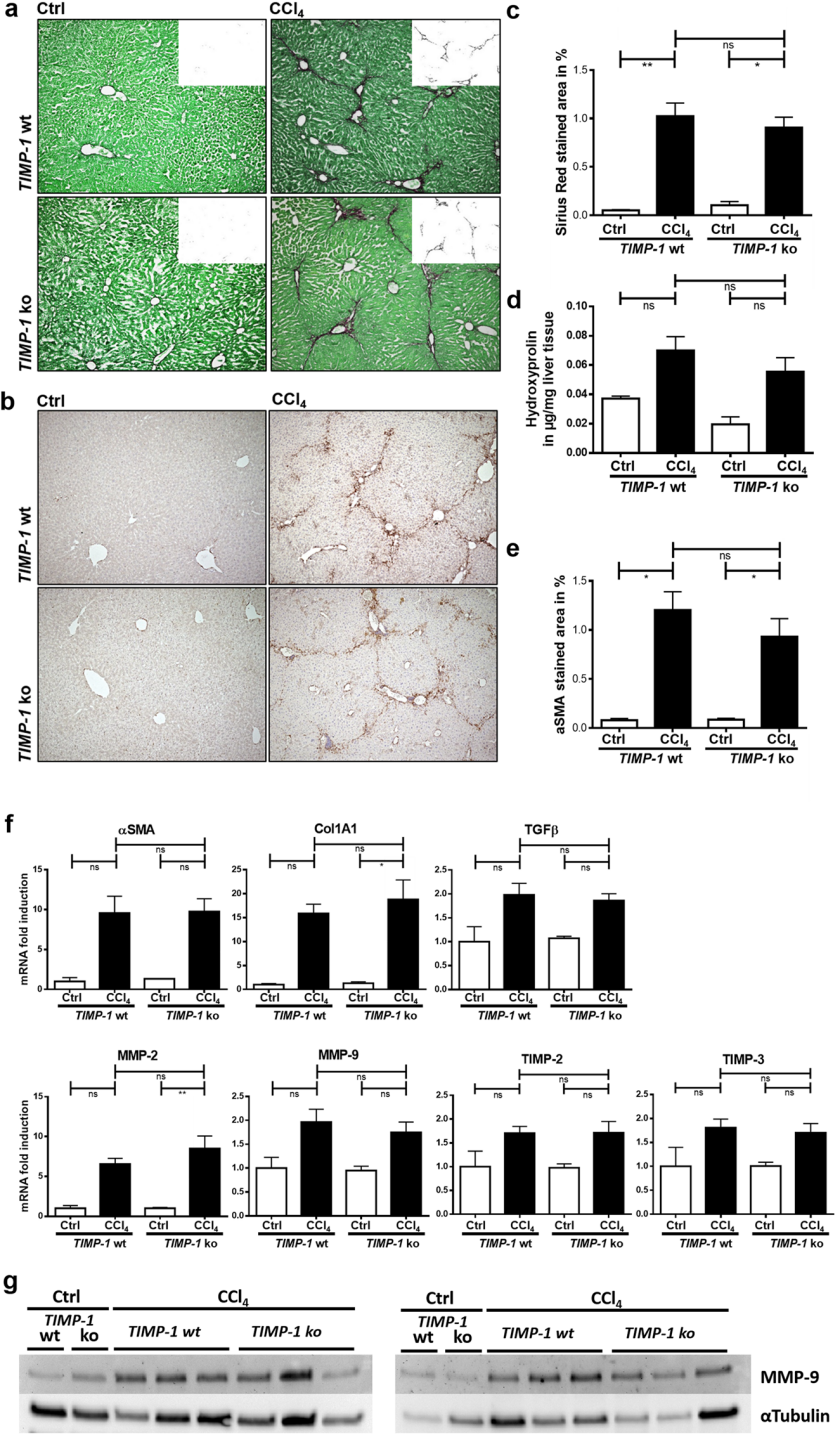
TIMP-1 deficiency does not prevent hepatic fibrogenesis following CCl_4_ treatment. (**a**) Collagen content of livers following chronic CCl_4_ treatment was assessed by Sirius Red staining. TIMP-1 deficiency does not alter collagen content compared to *TIMP-1* wt, as shown in representative images and (**c**) in quantification of Sirius Red positive stained area. (**d**) Supportively hepatic hydroxyproline content also did not differ between TIMP-1 deficient and wildtype animals following CCl_4_ treatment (**b**,**e**) αSMA IHC indicates hepatic stellate cell activation and was increased in livers following CCl_4_ administration. *TIMP-1* ko have the same amount of αSMA positive cells compared to *TIMP-1* wt. (**f**) Fibrosis-related genes were upregulated in livers after CCl_4_ treatment, but no differences could be observed between *TIMP-1* wt and *TIMP-1* ko mice. Not only MMP-9 gene expression was not altered, but (**g**) also Western blot analysis of MMP-9 protein showed no differences in *TIMP-1* ko livers compared to wildtypes. Displayed are cropped images. Full-length blots are presented in Supplementary Figure 3. Bar columns represent mean ± standard error of the mean. **p <  0.01; *p < 0.05; ns (non-significant). (Untreated controls n = 3–5, CCl_4_ n = 8–10).

To rule out that the lack of an effect of TIMP-1-deficiency on hepatic fibrogenesis is limited to cholestatic liver injury, we exposed wild type and *TIMP-1* ko mice to CCl_4_-induced liver injury as a second toxic model of hepatic fibrogenesis. Similar to the results in the BDL experiment, Sirius Red staining, αSMA immunohistochemistry, and qPCR of fibrosis-related genes showed no differences between *TIMP-1* ko mice and wild type littermates (Fig. 3a–f). Moreover, measurement of hepatic hydroxyproline content showed no significant difference between wild type and *TIMP-1 ko* mice that underwent BDL or CCl_4_ treatment, respectively (Fig. 3d).

Interestingly, mRNA expression of MMP-2 and MMP-9 was similar in wild type mice and *TIMP-1* ko mice, so TIMP-1-deficiency did not affect MMP expression (Fig. 3f). Moreover, Western blot did not show an upregulation of MMP-9 protein in *TIMP-1* ko mice (Fig. 3g). We did not observe a compensatory upregulation of other TIMP isoforms in *TIMP-1* ko mice in both models of fibrogenesis (Figs 2g and 3f).

In summary, our data do not support an essential function of TIMP-1 during hepatic fibrogenesis in mice.

### TIMP-1-deficient mice are not protected from hepatic carcinogenesis

TIMP-1 expression is upregulated in chronic liver injury and liver fibrosis, two conditions that precede the development of hepatocellular carcinoma (HCC). To determine the role of TIMP-1 in hepatic carcinogenesis, we treated wild type and TIMP-1 deficient mice with the liver carcinogen DEN. All *TIMP-1 ko* mice and wild type littermates had developed liver tumors at the age of 40 weeks.

**Figure 4:**
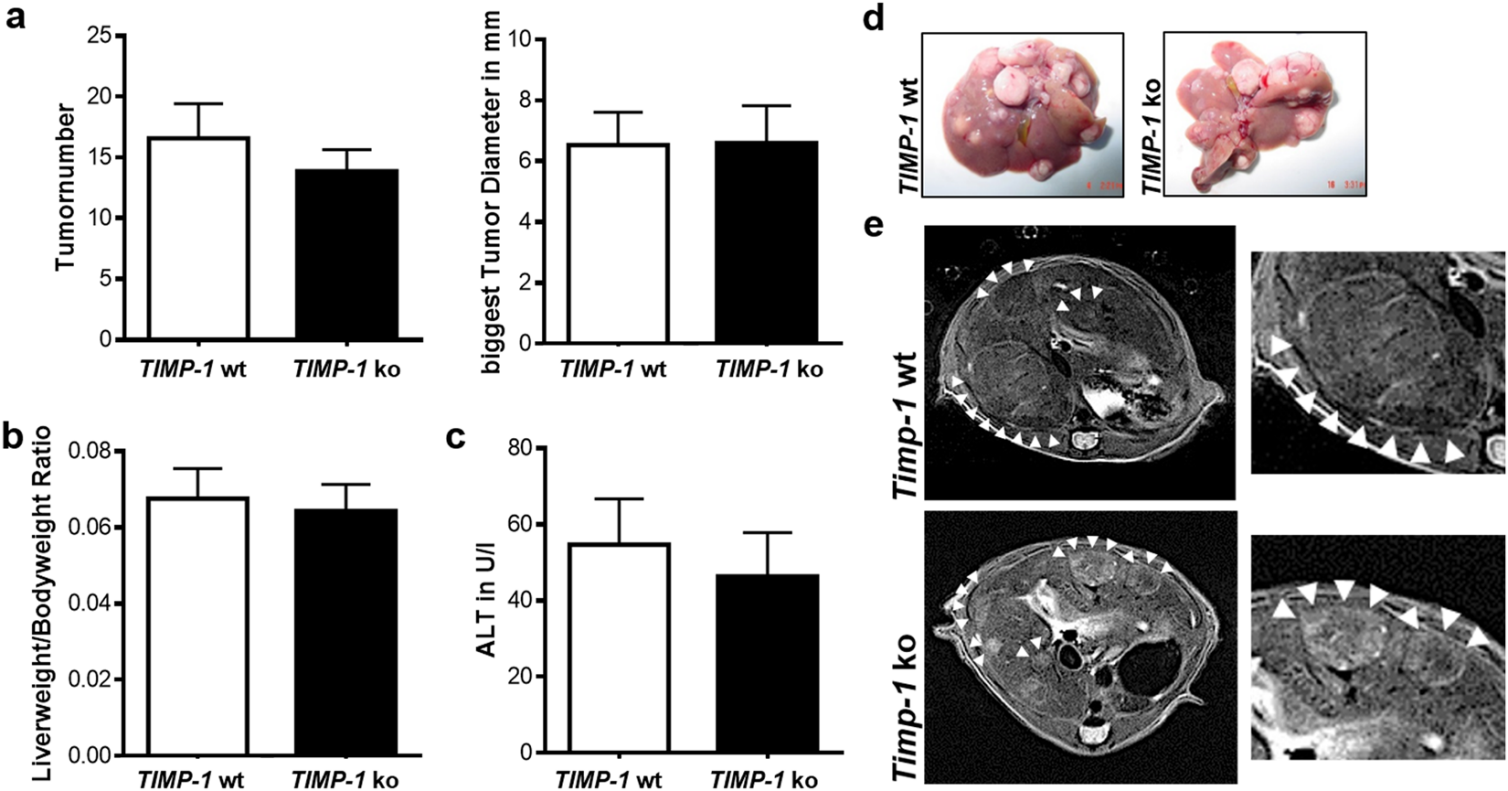
TIMP-1 deficient mice are not protected from hepatic carcinogenesis. Liver carcinogenesis was induced via juvenile DEN injection. (**a**) No differences between *TIMP-1* wt and ko could be observed in tumor number or size. (**b**) *TIMP-1* wt and ko show the same liverweight/bodyweight ratio. (**c**) ALT level were not induced following DEN treatment and did not differ between *TIMP-1* wt and *TIMP-1* ko. (**d**) Representative pictures of excised livers and (**e**) *in vivo* MRI images show equal tumor load of livers. Bar columns represent mean ± standard error of the mean. (n = 16–22).

Quantification of hepatic tumor load showed no difference between wild type and *TIMP-1* ko mice (Fig. 4a–e) and there was no difference in ALT levels (Fig. 4c).

**Figure 5:**
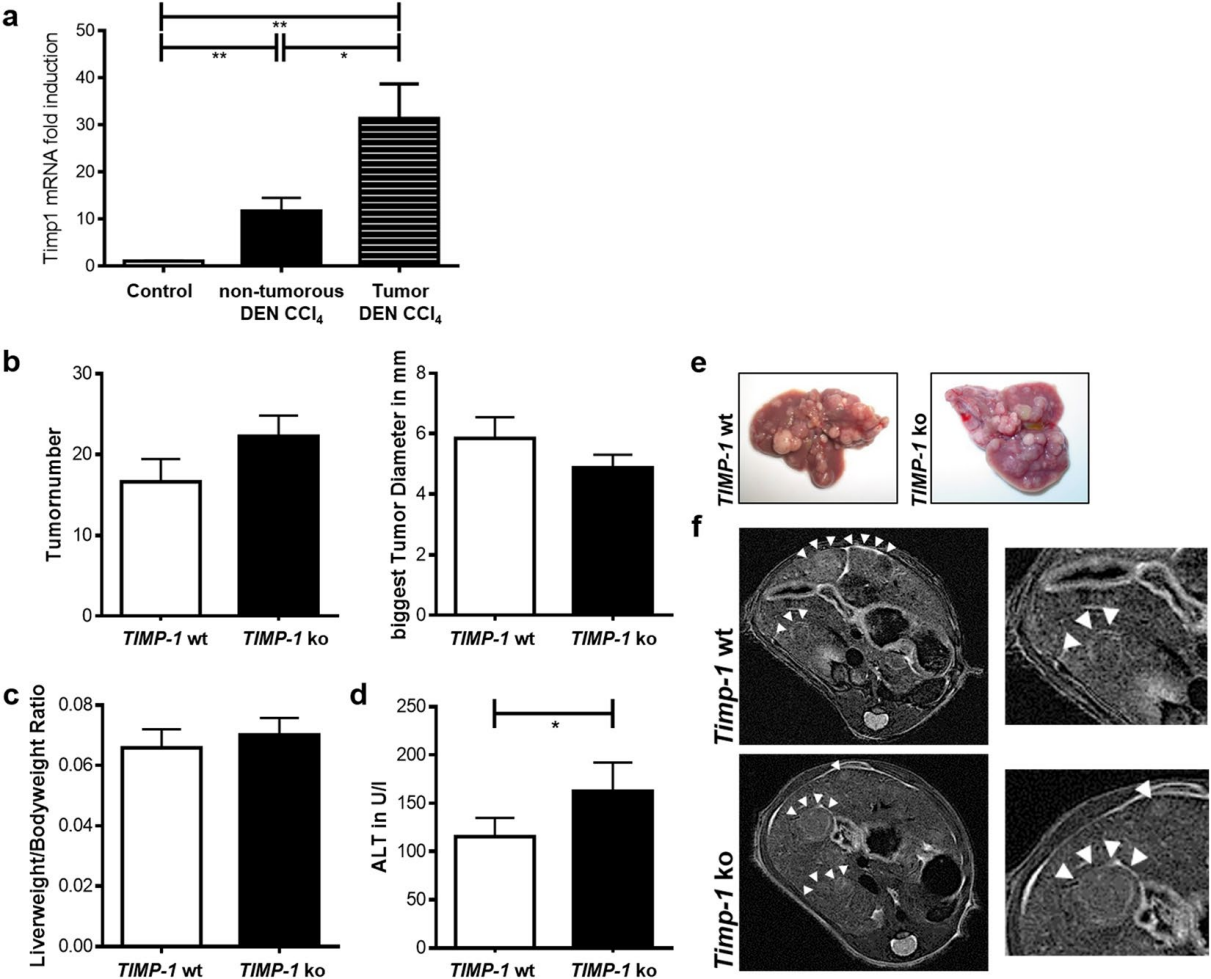
TIMP-1 deficient mice are not protected from fibrosis-driven hepatic carcinogenesis. (**a**) TIMP-1 expression increased in HCC tissue compared to adjacent paired normal tissue and control liver tissue following juvenile DEN administration and chronic CCl_4_ injection. (**b**) Tumorload does not differ between *TIMP-1 wt* and ko as assessed by tumor number and size and (**e**) visualized by representative pictures of excised livers and (**f**) *in vivo* MRI images. (**c**) TIMP-1-deficiency does not alter the liverweight/bodyweight ratio. (**d**) A combination of juvenile DEN administration and chronic CCl_4_ treatment led to overall increased ALT level, whereas elevated level could be measured in serum from *TIMP-1* ko mice as compared with *TIMP-1* wt. Bar columns represent mean ± standard error of the mean. **p <  0.01; *p < 0.05. (n = 13–19).

To test the hypothesis that TIMP-1 is relevant for fibrosis-driven tumor promotion in the liver we combined DEN-induced hepatocarcinogenesis with fibrogenic liver injury by CCl_4_. Interestingly, in CCl_4_/DEN-treated wild type mice TIMP-1-mRNA expression was significantly higher in liver tumor nodules than in surrounding non-tumorous liver tissue (Fig. 5a). However, quantification of hepatic tumor load as assessed by tumor number, size, liver-to-body-weight-ratio and MRI revealed no significant differences between *TIMP-1 ko* mice and their wild type littermates (Fig. 5b–f). ALT levels as a marker of hepatocellular injury were significantly higher in *TIMP-1* ko mice (Fig. 5d).

Thus, TIMP-1-deficient mice are not protected from the development of hepatocellular carcinoma.

## Discussion

Both, profibrogenic and carcinogenic functions have been attributed to TIMP-1 and TIMP-1 expression levels have been shown to correlate with liver fibrosis and presence of hepatocellular carcinoma^5–8, 25^. Thus, TIMP-1 could represent the missing functional link for the unique association of fibrogenesis and carcinogenesis in chronic liver disease and thereby a promising therapeutic target to prevent not only progression of liver disease but also the development of HCC. Here, we study the functional relevance of TIMP-1 in the development of liver fibrosis and hepatocellular carcinoma *in vivo*.

Two animal studies that used anti-TIMP-1 antibodies^13^ and a matrix metalloproteinase 9 (MMP-9) mutant as a TIMP-1 scavenger^12^ to antagonize TIMP-1 suggested that TIMP-1 inhibition attenuates hepatic fibrogenesis. Contrasting these data, our experiments using mice with genetic *TIMP-1*-deletion did not show an essential contribution of TIMP-1 in the development of liver fibrosis while we could confirm that *TIMP-1 ko* mice in fact lack TIMP-1 protein. One could speculate that both anti-TIMP-1 protein strategies could exert pleiotropic antifibrotic effects that are not directed against TIMP-1 specifically, e.g. by binding other TIMP-isoforms. Another possible explanation could be that in TIMP-1-deficient mice compensatory mechanisms replace profibrogenic functions of TIMP-1, so that TIMP-1 might indeed promote hepatic fibrogenesis but is not indispensable. This could also explain the discrepancy between our data and studies that have demonstrated that transgenic TIMP-1 overexpression promoted hepatic fibrogenesis and delayed fibrosis reversal^10, 11^. Contrasting these data that suggest profibrogenic functions for TIMP-1, a recent study found that TIMP-1-deficient mice were even more susceptible to CCl_4_-induced liver fibrosis than wild type mice^14^. Consistent with this, we observed a higher collagen I mRNA expression in *TIMP-1 ko* mice in both fibrosis models, BDL and CCl_4_, while our histological fibrosis assessment did not show a difference between wild type and *TIMP-1 ko* mice. A possible explanation for divergent results in the study by Wang *et al*. and our CCl_4_ experiment could be that Wang *et al*. used non-littermate wild type mice as controls whereas we used littermate controls. Moreover, in the study by Wang *et al*. a higher dose of CCl_4_ was applied than in our study. The authors claim that the antifibrotic net effect of TIMP-1 observed in their study results from the prevention of hepatocellular injury which outweighs a potential profibrogenic function of TIMP-1^14^. One could speculate that, with the higher CCl_4_ dose, TIMP-1-dependent hepatoprotection is more relevant than the profibrogenic effects of TIMP-1. Our findings however, do not support specific targeting of TIMP-1 as an antifibrotic therapeutic strategy.

Previous studies have not only attributed antiapoptotic and proangiogenic functions to TIMP-1^16, 19^ but also established serum TIMP-1 as a marker of poor prognosis in extrahepatic malignancy^20–24^. Interestingly, a recent study demonstrated that treatment of mice with recombinant TIMP-1 or adenoviral delivery of TIMP-1 increased homing of tumor cells to the liver and formation of hepatic metastasis by creating a premetastatic niche^30^. Consistent with our findings in murine HCC, in human hepatocellular carcinoma, TIMP-1 expression is significantly higher than in non-neoplastic liver^26, 31^. In addition, high TIMP-1 serum levels indicated presence of HCC in patients in one study^25^. In view of this strong association of TIMP-1 expression not only with fibrogenic liver injury but also with malignancy, we hypothesized that TIMP-1 would functionally promote hepatic carcinogenesis as a relevant part of the cirrhotic microenvironment. However, our data clearly show that TIMP-1-deficiency does not attenuate DEN-induced hepatic carcinogenesis in mice. One major weakness of the DEN model of hepatic carcinogenesis used in our study is that it does not reproduce the phenotype with chronic liver inflammation and fibrogenesis that is present in the majority of patients who develop HCCs. Thus, a merely genotoxic carcinogenesis model such as DEN could fail to reproduce potential tumor promoting effects of TIMP-1 which is upregulated during chronic liver fibrogenesis. However, even when we combined the genotoxic carcinogen DEN with induction of liver fibrosis by CCl_4_, liver tumor growth in TIMP-1-deficient mice and their wild type littermates was similar. Therefore, we could not confirm our hypothesis that TIMP-1 mediates fibrosis-induced promotion of HCC. In our mouse study TIMP-1 has no functional relevance for HCC progression.

This does not contradict the observation in HCC patients that higher TIMP-1 expression is correlated with a poorer post-resection survival as in this study the group with high TIMP-1 expression also had more advanced malignancy^26^ and also higher TIMP-1 expression was correlated with lower grade of tumor differentiation^26, 31^. Thus, increased TIMP-1 expression could be a progression marker in HCC development, indicating late tumor stages and dedifferentiation, rather than being a functional mediator of tumor initiation or promotion.

In summary, our data are consistent with published evidence that TIMP-1 is upregulated in liver fibrosis and hepatocellular carcinoma, potentially implying diagnostic relevance in the non-invasive assessment of liver fibrosis or in HCC detection^6, 7, 9, 25^. However, our study in TIMP-1-deficient mice did not confirm a functional role of TIMP-1 in the development of liver fibrosis or hepatocellular carcinoma.

## Materials and Methods

### Mice, induction of liver fibrosis and hepatic tumorigenesis

TIMP-1-deficient (Timp1^tm1Pds^/J) mice (*TIMP-1* ko mice) were purchased from Charles River and kept in the animal facility of the Hamburg University Medical Center under specific pathogen-free conditions. Animal care was in accordance with the governmental and institutional guidelines and all experiments were approved by the animal experimentation committee of the State of Hamburg (G10/082, G16/100). Hepatic fibrosis was induced in 16–23 weeks old male *TIMP-1* ko mice (n = 5–9) or wild type (wt) (n = 9–11) littermates by ligation of the common bile duct (BDL) for 13 days as described previously^4, 32^ or by intraperitoneal injections (i.p.) of carbon tetrachloride (CCl_4_, Merck, Darmstadt, DE, 0.5 µl/g body weight (bw), diluted 1:4 in corn oil, Sigma-Aldrich, St. Louis, MO, USA) twice per week over 2 to 6 weeks.

HCC formation was induced in male *TIMP-1 ko* mice (n = 21) or wild type littermates (n = 16) by intraperitoneal injection of diethylnitrosamine (DEN, Sigma-Aldrich, St. Louis, MO, USA, 25 mg/kg)^33^ on day 16 postpartum. Mice were sacrificed at the age of 40 weeks. For combined induction of liver fibrosis and HCC, 15 days-old male *TIMP-1 ko* (n = 19) mice or wild type littermates (n = 13) received intraperitoneal injections of DEN (25 mg/kg) followed by weekly intraperitoneal CCl_4_ injections starting at the age of 8 weeks. These mice were sacrificed after 20 weeks of CCl_4_ treatment.

### Histology

Sections of formalin-fixed and paraffin-embedded liver tissue were stained with Sirius Red for fibrillary collagen. The Sirius Red-positive area was quantified in at least 10 low power fields using Adobe Photoshop and ImageJ in a blinded fashion. Detection of activated HSCs in paraffin-embedded liver sections was achieved by α-smooth muscle actin-immunostaining (anti-αSMA, ab5694, Abcam, Cambridge, UK). Hepatic macrophages were stained using an anti-F4/80 antibody (clone CI:A3-1, BioLegend, San Diego, CA).

### Quantification of hepatic tumor load

Tumor load and time point of tumorigenesis was assessed *in vivo* by magnetic resonance imaging (MRI) using a small animal system at 7 Tesla (ClinScan, Bruker, Ettlingen, DE). Mice were anaesthetized via inhalation of 2% isoflurane, 98% oxygen at a flow rate of 500 ml/min. Tumor load was also quantified *ex vivo* by counting the number of visible tumors and measuring the size of the largest tumor with a caliper^33^.

### Mouse HSC isolation, culture and Western blot analysis

HSCs were isolated from normal mouse livers or from mouse livers 48 h after fibrogenic injury with CCl_4_ by pronase-collagenase perfusion followed by Nycodenz-based density centrifugation as described previously^4^. For *in vivo* activation of HSCs mice received four i.p. injections of CCl_4_ (0.5 µl/g bw) at intervals of three days. Following isolation, HSCs were plated in Dulbecco’s modified Eagle medium (DMEM) containing 10% fetal bovine serum (FBS), glutamine, HEPES buffer, and antibiotics.

HSCs were lysed after 16 hours in culture. Liver tissue was also lysed in RIPA buffer (20 mM Tris; 1% Triton X; 2.5 mM Sodiumpyrophosphate; 150 mM NaCl; 1 mM EGTA; 1 mM EDTA) containing 2 mM Na_3_VO_4_, 10 mM NaF and a protease inhibitor cocktail (Roche, Basel, CH) for further analysis. Protein concentrations were quantified with a BCA protein assay kit (Pierce, Rockford, IL) according to the manufacturer’s manual. Protein extracts were denatured in loading buffer, separated by SDS-PAGE using 15% gels according to Laemmli^34^ and transferred to a nitrocellulose membrane (Merck Millipore, Darmstadt, DE). Membranes were incubated with antibodies to TIMP-1 and MMP-9 (R&D Systems, Minneapolis, MN), αSMA (Sigma-Aldrich, St. Louis, MO, USA) and, as a loading control, total actin or αTubulin (Santa Cruz, Dallas, TX, USA) followed by incubation with horseradish peroxidase-conjugated secondary antibodies and enhanced chemoluminescence detection with ChemiDoc (Bio-Rad, Hercules, CA, USA).

### ﻿Measurement of hepatic hydroxyproline content

Hydroxyproline assay was performed as described previously^35﻿^.

### ﻿Measurement of Plasma alanine-aminotransferase

Liver damage was assessed by measuring plasma enzyme activity of alanine aminotransferase (ALT) using an automated procedure (COBAS MIRA; Roche, Basel, Switzerland).

### Real-time PCR

RNA was isolated from whole liver tissue using the RNeasy Kit (Macherey-Nagel, Düren, DE) and reverse transcribed (cDNA Synthesis Kit, Life Technologies, Carlsbad, CA USA) according to the manufacturer’s recommendations. For tumor experiments, tissue from HCC and non-tumorous liver was separated to allow for comparative expression analyses.

Quantitative real-time PCR (qPCR) was performed with the Viia7 System and primer probe sets from Life Technologies, Carlsbad, CA). Data were normalized to 18 s and quantified using a relative standard curve. Primers used were: 18 s 4319413E, αSMA Mm01546133_m, Col1A1 Mm00801666_g1, TGFβ Mm03024053_m1, MMP-2 Mm00439498_m1, MMP-9 Mm00442991_m1, TIMP-2 Mm00441825_m1, TIMP-3 Mm00441826_m1.

### Statistical analysis

All data are expressed as mean ± SEM. Statistical analysis was performed by the two-tailed unpaired Student t test or by one-way ANOVA with Tukeys post hoc test. Differences were considered significant at p values < 0.05.

## Electronic supplementary material

Supplementary Figure 1, 2, 3
