# Supplementary material for: TIMP-1 is upregulated, but not essential in hepatic fibrogenesis and carcinogenesis in mice
Source: Sci Rep. 2017 Apr 6;7:714. doi: 10.1038/s41598-017-00671-1 (PMC5428806; doi:10.1038/s41598-017-00671-1)
Supplement: Supplementary file 1 — Supplementary Figure 1, 2, 3 [file 41598_2017_671_MOESM1_ESM.doc]

**Title:**

**TIMP-1 is upregulated, but not essential in hepatic fibrogenesis and carcinogenesis in mice**

**Authors**:

Nina D. Thiele*, Jan W. Wirth*, David Steins, Anja C. Koop, Harald Ittrich, Ansgar W. Lohse, Johannes Kluwe

Nina D. Thiele, Jan W. Wirth, David Steins, Anja C. Koop, Ansgar W. Lohse, Johannes Kluwe: I. Department of Internal Medicine, University Medical Center Hamburg-Eppendorf, Hamburg, Germany

Harald Ittrich: Department of Diagnostic and Interventional Radiology and Nuclear Medicine, University Medical Center Hamburg-Eppendorf, Hamburg, Germany

*These authors contributed equally.

**Grant support:** This work was supported by the German Research Foundation (grants SFB841 to H.I., A.W.L. and J.K. and KL2140 to J.K.) and by the European Association for the Study of Liver Diseases (Sheila Sherlock Short-Term Fellowship to J.K.)

**Competing financial interests:** The authors declare no competing financial interests.

**Author contributions statement:**

Nina D. Thiele assisted in animal experiments, performed cell isolations, real-time PCRs, Western blots, immunohistochemistry, contributed to experimental setup, data analysis and interpretation and assisted in manuscript preparation.

Jan W. Wirth assisted in animal experiments, performed fibrosis evaluation, real-time PCR, immunohistochemistry, contributed to experimental setup, data analysis and interpretation and critically revised the manuscript.

David Steins helped with fibrosis evaluation and immunostaining.

Anja C. Koop performed and evaluated immunostaining.

Harald Ittrich performed animal MRIs.

Ansgar W. Lohse critically revised the manuscript.

Johannes Kluwe designed and coordinated the study, performed animal experiments, contributed to experimental setup, data analysis and interpretation, drafted and edited the manuscript.


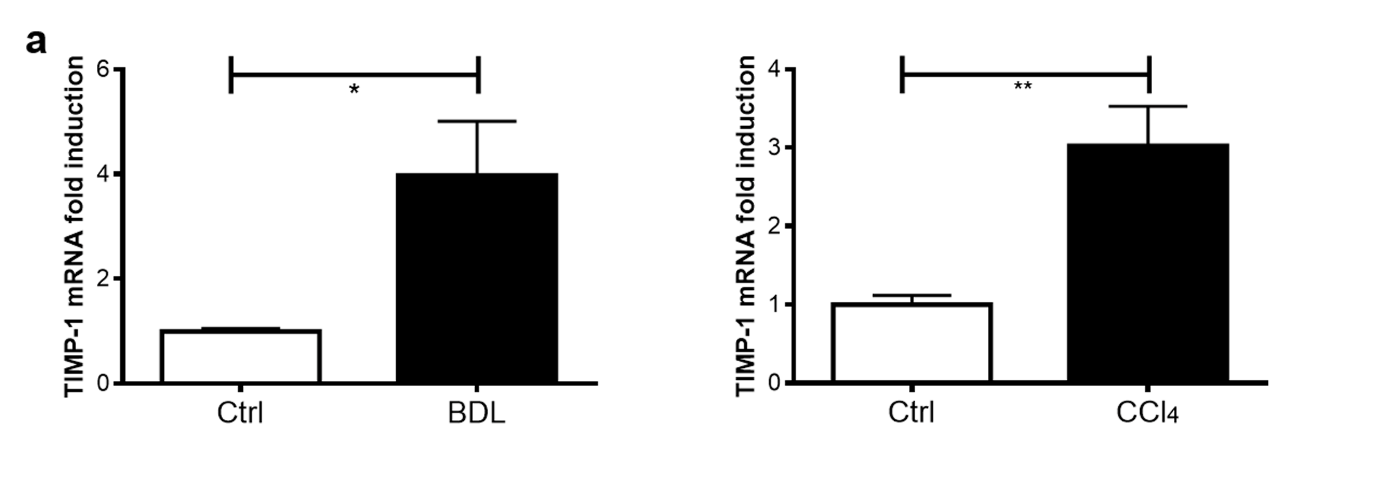


Supplementary Figure 1: **TIMP-1 upregulation in chronic models of liver fibrosis**

(**a**) Hepatic TIMP-1 expression is upregulated in chronic models of liver fibrosis, such as bile duct ligation (BDL) (left panel) and CCl4 treatment (right panel). Bar columns represent mean ± standard error of the mean. **p <  0.01; *p < 0.05. (Untreated controls n=3, treatment n=9)


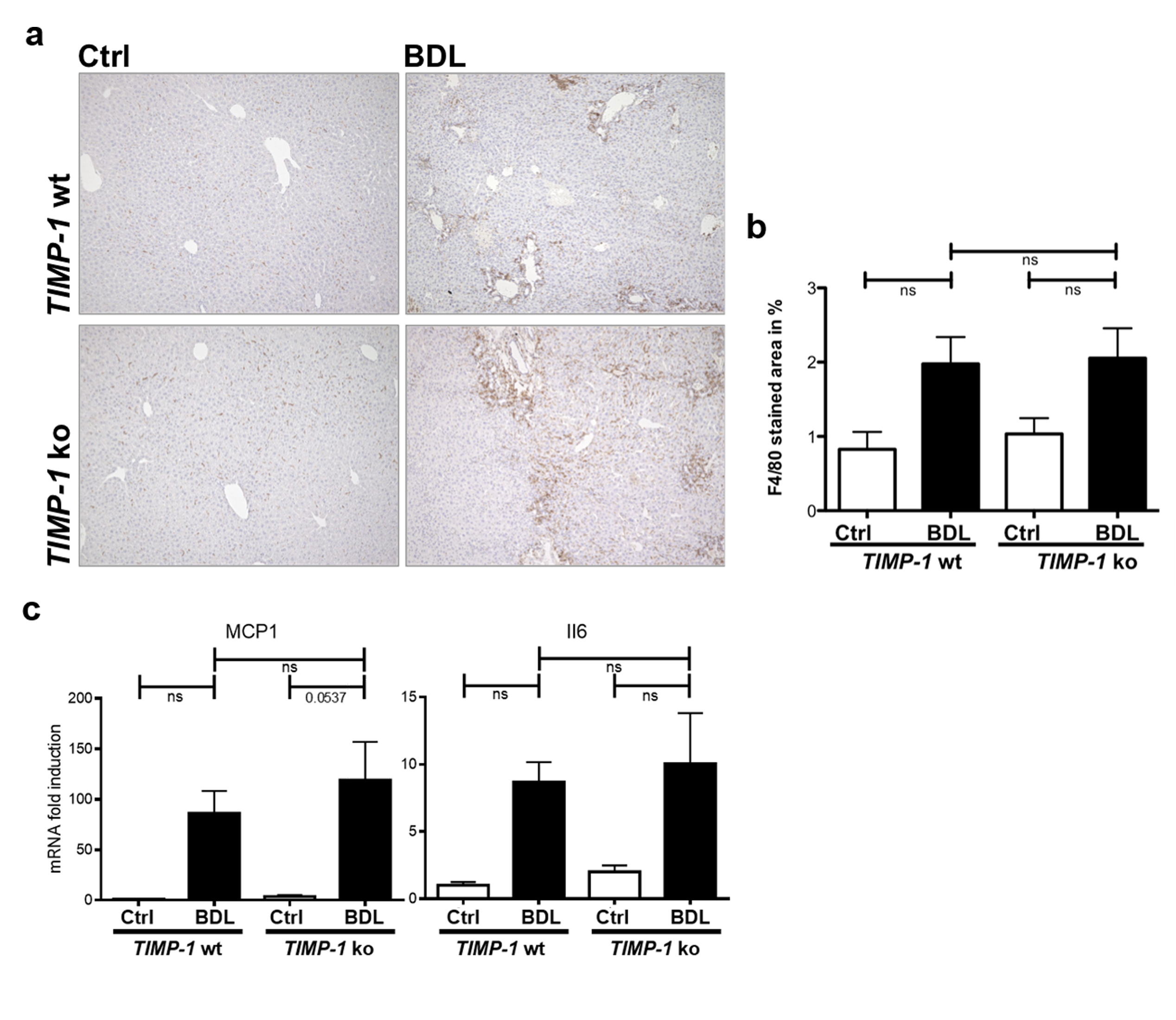
Supplementary Figure 2: **TIMP-1 deficiency does not prevent hepatic inflammation in the BDL model**

(**a, b**) Hepatic infiltration of F4/80 positive cells is increased following bile duct ligation (BDL) but is not altered in *TIMP-1* ko compared to *TIMP-1* wt. (**c**) Expression of proinflammatory genes such as MCP1 and Il6 is highly induced in bile duct ligated livers but does not differ between *TIMP-1* ko and *TIMP-1* wt. Bar columns represent mean ± standard error of the mean. ns (non-significant). (Untreated controls n=2-4, BDL n=5-11)


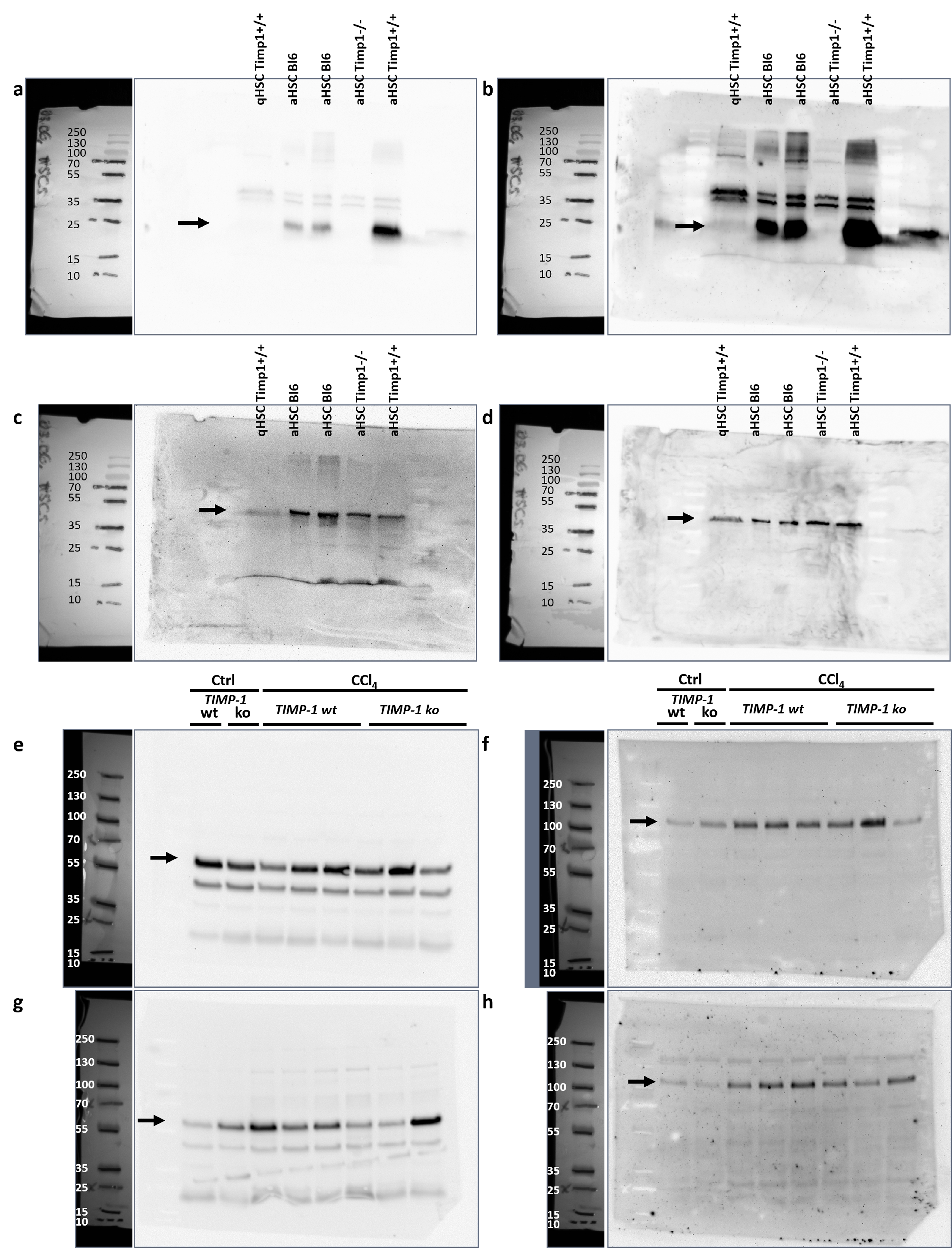


Supplementary Figure 3: **Full-length blots of relevant proteins**

(**a**) TIMP-1 10 seconds exposure, (**b**) TIMP-1 30 minutes exposure, (**c**) αSMA, (**d**) Actin, (**e, g**) αTubulin, (**f, h**) MMP-9
